# Pharmaco-Electroencephalography-Based Assessment of Antidepressant Drug Efficacy—The Use of Magnesium Ions in the Treatment of Depression

**DOI:** 10.3390/jcm10143135

**Published:** 2021-07-15

**Authors:** Michał Skalski, Anna Mach, Piotr Januszko, Beata Ryszewska-Pokraśniewicz, Agata Biernacka, Gabriel Nowak, Andrzej Pilc, Ewa Poleszak, Maria Radziwoń-Zaleska

**Affiliations:** 1 Department of Psychiatry, Medical University of Warsaw, 00-665 Warsaw, Poland; michal.skalski@wum.edu.pl (M.S.); piotr.januszko@wum.edu.pl (P.J.); maria.radziwon@wum.edu.pl (M.R.-Z.); 2 Mazowieckie Centrum Neuropsychiatrii, 05-462 Wiązowna, Poland; beataryszewska@wp.pl; 3 Private Practice, 02-591 Warsaw, Poland; dragatabiernacka@gmail.com; 4 May Institute of Pharmacology, Polish Academy of Sciences, 31-343 Kraków, Poland; gabriel.nowak@uj.edu.pl (G.N.); nfpilc@cyf-kr.edu.pl (A.P.); 5 Faculty of Pharmacy, Medical University of Lublin, 20-093 Lublin, Poland; ewa.poleszak@umlub.pl

**Keywords:** unipolar depression, magnesium, pharmaco-electroencephalography, efficacy, remission

## Abstract

Pharmaco-electroencephalography (pharmaco-EEG) is a technique used to assess the effects of psychotropic medications on the bioelectrical activity of the brain. The purpose of this study was to assess the treatment response with the use of the Hamilton Depression Rating Scale (HDRS) and via EEG. Over an 8-week period, we analyzed electroencephalographic tracings of 91 patients hospitalized for major depression at the Medical University of Warsaw. Thirty-nine of those patients received tricyclic antidepressants (TCAs), 35 received fluoxetine, and 17 received fluoxetine augmented with magnesium (Mg) ions. All patients had their serum drug levels monitored. The highest proportion of patients (88.2%) who showed adequate responses to treatment was observed in the fluoxetine+Mg group, whereas the lowest rates of treatment response were observed in the TCA group (58.3%). This difference was statistically significant (*p* = 0.029, Phi = 0.30). Our study demonstrated a relationship between achieving remission (HDRS ≤ 6 at week 8 of treatment) and obtaining a positive pharmaco-EEG profile 6 h after administration of the first dose in the group receiving fluoxetine augmented with Mg ions (*p* = 0.035, Phi = 0.63).

## 1. Introduction

Major depressive disorder is currently the fourth leading cause of disease-specific premature death [1,2], and its social and economic burden is comparable with that of cancer and cardiovascular disease.

World Health Organization projections estimate that recurrent depressive disorder will rank first among diseases carrying the risk of premature death and loss of income due to disability (characterized by disability-adjusted life years (DALY)) by the year 2030 [3].

One-third of patients with severe recurrent depressive disorder fail to improve after the first antidepressant regimen.

“Nearly all currently available antidepressants lead to clinical improvement in 50–70% of patients; the aim of antidepressant treatment should be to achieve recovery. In the case of psychiatric disorders, including depression, ‘recovery’ is defined as ‘remission’, i.e., a symptom-free period” [4].

First-line antidepressant treatment helps achieve remission in 25–35% of patients.

Magnesium, zinc, and copper are NMDA receptor modulators. Recent data implicate a disrupted NMDA receptor-dependent glutamatergic transmission in the pathophysiology of depression [5].

In light of the mechanism of action of magnesium ions and their suspected role in the glutamate hypothesis of depression, attempts have been made to augment antidepressant treatment with magnesium supplementation [6].

Animal studies demonstrate that zinc and magnesium supplementations improve the effectiveness of antidepressant treatment [5,7]. The antidepressant effects of zinc and magnesium have been discussed in the context of their impact on the NMDA receptor complex, brain-derived neurotrophic factor (BDNF), and glycogen synthase kinase 3 (GSK-3) [5].

Pharmaco-electroencephalography (pharmaco-EEG) has been used to evaluate the effect of drugs on the bioelectrical activity of the brain [8,9,10].

The basic assumption behind performing pharmaco-EEG is the fact that all chemical compounds that affect behavior also modify electrocardiographic patterns [11,12,13,14], and the directionality of electrocardiographic changes depends on the clinical effects of the given drug. Drug-induced changes in EEG are the same in healthy individuals and in patients suffering from an illness, and short-term changes in EEG are predictive of changes occurring during long-term therapy [11,15,16,17].

Several typical pharmaco-EEG profiles have been identified [11,18]:

In response to anxiolytics, EEG tracings show increased low-frequency beta (beta 1) activity, decreased power of alpha rhythm, and increased power of delta and theta oscillations.

Antipsychotic drugs decrease the power of theta and delta rhythms, decrease the power of beta 1 oscillations, and lower the frequency and power of the alpha rhythm.

Antidepressants cause a lower alpha frequency, and increased theta and high-frequency beta (beta 2) oscillations.

Stimulants (amphetamine-like drugs) decrease the power of delta, increase the power of alpha and beta, and reduce the power of theta oscillations.

Nootropic drugs lead to a reduced power of delta and theta rhythms and to increased alpha oscillations.

Lithium increases the power of delta and theta oscillations and slows the alfa rhythm.

Studies [15] have shown a correlation between serum TCA levels and alterations in the pharmaco-EEG patterns of beta-band (beta 2 and 3) and alpha 1 oscillations.

The character of pharmaco-EEG profiles observed over several weeks of treatment did not markedly differ from the pharmaco-EEG profiles of TCAs presented in earlier literature reports (increased high- and low-frequency beta activity, and decreased middle-range activity) from studies involving healthy individuals participating in several-hour-long experiments.

In his studies from the 1980s and 1990s, Saletu attempted to divide selective serotonin reuptake inhibitors (SSRIs) (such as TCAs) into thymoleptic drugs (of imipramine- and amitriptyline-like activity), with this group including. 

The differences between the pharmaco-EEG profiles of patients with adequate fluvoxamine [16,17], venlafaxine [18], and sertraline at >100 mg [19], and tachythymoleptic drugs (of desipramine-like activity), such as fluoxetine [16,20] and sertraline at <100 mg [19].and inadequate responses to treatment indicate that the appearance of an antidepressant EEG profile may have a significant predictive value [15,21].

Previous study results [11,22,23,24,25,26,27,28] give us hope for the usefulness of pharmaco-EEG as a tool for assessing the response to treatment.

**Purpose of this study:** The purpose of this study was to assess the treatment response with the use of the Hamilton Depression Rating Scale (HDRS) and via EEG.

## 2. Materials and Methods

The study evaluated 91 patients treated at the Department of Psychiatry Medical University of Warsaw and the Nowowiejski Hospital in Warsaw in the period between 1998 and 2017 who met the ICD-10 criteria of a depressive episode or the DSM-IV criteria of major depression. All patients were treated in a psychiatric ward. Consecutively admitted patients were included in the study, provided they met the inclusion criterion of a normal baseline EEG pattern. Patients with the following conditions were excluded from the study: delusional disorders and organic disorders, high risk of suicide requiring the use of electroconvulsive therapy, absolute contraindications for treatment with TCAs or SSRIs, absolute contraindications for magnesium supplementation, drug or alcohol abuse, and baseline EEG abnormalities.

There was no age limit or restrictions as to the patients’ sex.

The study was approved by the Institutional Review Board of Medical Academy of Warsaw (KB 95/1998; KB 42/2005; KB 96/2006) before and after (KB 227/2012) it was renamed as the Medical University of Warsaw.

Study participation was voluntary and required signing informed consent.

In line with the review board’s protocol, patients signed informed consent prior to their participation in the study.

The patients were assessed for qualification for participation based on their baseline assessments, which included a clinical examination, psychometric scale scores, and EEG. After inclusion in the study, the patients underwent a 1–2-week wash-out period. In patients treated with SSRIs, the wash-out period was extended to 6 weeks.

Study subjects were examined by the same physician at consecutive time points: prior to treatment initiation (at baseline); 6 h after the first dose (maximum blood levels after the first dose); at 24 h; and 2, 4, 6, and 8 weeks after the first dose. The preliminary results of this study were reported elsewhere [15,24,26,27].

The psychometric tool used in this study was the 21-item version of the Hamilton Depression Rating Scale (HDRS). A 50% reduction in the baseline HDRS score was adopted as the cutoff point for treatment response (effectiveness).

Remission was defined as achieving an HDRS score of 6 or less.

During the study, the patients were advised to avoid taking any drugs that could affect the levels of the evaluated antidepressant.

Standard dosing regimens were used in all study groups. Antidepressant and magnesium blood levels were monitored and maintained within the therapeutic range [24,27].

Seventeen patients had their standard fluoxetine regimen augmented with magnesium. Magnesium aspartate was administered in the form of Magnesium Effervescent or a powder containing 40 mg of magnesium (equivalent to 3.30 mEq of magnesium) at a dose of 40 mg three times a day.

TCA and fluoxetine blood levels were measured at the psychopharmacological laboratory of the Department of Psychiatry.

Serum magnesium levels were measured at an Alab laboratory, with the use of Hulanicki’s technique [29].

EEG was performed prior to treatment initiation; 6 and 24 h after the first dose; and subsequently at treatment weeks 2, 4, 6, and 8.

EEG examinations involved 10-min recordings from four montages: F3–C3, F4–C4, P3–O1, and P4–O2 (according to the 10–20 international system of electrode placement). The filter setup used a band-pass of 0.16–40.0 Hz. In order to exclude artifacts, the EEG tracings were evaluated visually in 2.5-s segments.

EEG assessments were performed with a DigiTrack DTW electroencephalograph (Elmico). Subsequently, the relative power of the electroencephalographic signal was calculated with NeuroGuide software and a fast Fourier transform (FFT) algorithm.

Adopting a 0.5-Hz resolution, we calculated the power spectra in the delta (1.5–5.0 Hz), theta (5.5–8.0 Hz), alpha 1 (8.5–10.0 Hz), alpha 2 (10.5–12.0 Hz), beta 1 (12.5–18.5 Hz), beta 2 (19.0–20.5 Hz), and beta 3 (21.0–29.5 Hz) frequency bands.

Arranged chronologically, t-test values for the individual bands formed a profile of EEG power spectrum changes at each time point over the treatment period with respect to the baseline EEG pattern obtained prior to treatment initiation.

Each of the graphs was classified by an expert based on the presence or absence of an antidepressant-induced pharmaco-EEG profile. The pharmaco-EEG profile in fluoxetine-treated patients was considered positive when it exhibited TCA-like changes, namely an increase in high-frequency beta waves (beta 3) [11,15,23,25].

All EEG examinations were performed at the Clinical Electroencephalography and Neurophysiology Laboratory of the WUM Department of Psychiatry.

The study results were analyzed with descriptive statistics, such as the means, standard deviations, medians, and ranges. The Shapiro–Wilk test was used to test the null-hypothesis stating that the analyzed quantitative variables were normally distributed. If the distribution of a given variable was normal, the F-test was used to determine if any of the groups had the same mean (one-way analysis of variance (one-way ANOVA)). The groups in which the evaluated variables exhibited a distribution different from normal were compared with the Kruskal–Wallis test for independent samples. Associations between quantitative variables were evaluated with the chi-squared test or Fisher’s exact test for small frequencies in contingency table cells, and the measure of effect size was the Phi coefficient.

The level of statistical significance was adopted at a *p*-value of < 0.05*.

All calculations were conducted with the SAS 15.1 system.

## 3. Results

The group of 39 patients (42.9%) who received TCAs for 8 weeks consisted of 29 women and 10 men aged 52.0 ± 14.4 years (median 54.0; range 23.0–78.0), the mean duration of their disease was 10.9 ± 11.8 years (median 5.0; range 0.2–40.0), and the mean BMI was 24.4 ± 4.8 (median 24; range 15.2–33.6).

The group of 35 patients (38.5%) who received fluoxetine comprised 19 women and 16 men aged 51.5 ± 12.9 years (median 52.0; range 24.0–75.0), mean disease duration was 6.2 ± 7.1 years (median 4.0; range 0.4–26.0), and the mean BMI was 26.5 ± 5.5 (median 25.4; range 17.6–40.0).

Seventeen patients (18.7%), 11 of whom were women and 6 were men, received fluoxetine and Mg ions (fluoxetine + Mg). The mean age in this group was 48.1 ± 15.5 years (median 50.0; range 23.0–71.0), mean disease duration was 5.6 ± 5.8 years (median 4.0; range 0.3–20.0), and the mean BMI was 24.6 ± 4.4 (median 24.2; range 19.1–35.5).

The groups treated with fluoxetine, fluoxetine+Mg, and TCAs showed no significant differences in terms of age (*p* = 0.657), BMI (*p* = 0.238), disease duration (*p* = 0.174), or distribution of the sexes (*p* = 0.186).

The mean baseline HDRS score for the severity of depression symptoms in the TCA group was 27.0 ± 4.8 (median 25.0; ranging from 20.0 to 38.0).

In the fluoxetine group, the mean severity of depression symptoms measured via the HDRS before treatment initiation was 28.4 ± 5.5 (median 29.0; range 18.0–43.0).

The mean baseline HDRS score for the severity of depression in the fluoxetine + Mg group was 30.5 ± 6.0 (median 29.0; range 21.0–44.0).

After 8 weeks of treatment, the mean HDRS depression severity score in the TCA group was 12.9 ± 7.9 (median 10.0; range from 1.0 to 30.0).

In the fluoxetine group, the mean depression severity rated with the HDRS after treatment completion was 11.8 ± 8.0 (median 12.0; range from 1.0 to 39.0).

In the fluoxetine + Mg group, the mean HDRS depression severity score after 8 weeks of treatment was 10.7 ± 7.9 (median 8.0; range from 1.0 to 29.0).

Adequate treatment response (effectiveness), defined as a 50% improvement in the HDRS score after 8 weeks of treatment, was observed in 21 patients (58.3%) from the TCA group, in 20 patients (74.1%) from the fluoxetine group, and in 15 patients (88.2%) from the fluoxetine + Mg group.

There was no significant difference between the groups in the response to treatment after 8 weeks of therapy (*p* = 0.073). Due to the observed differences in treatment response within the individual groups, we conducted a less conservative post hoc comparison of selected parameters, which did not require rejecting the hypothesis of equal responses in all treatment groups.

The highest rate of adequate treatment response was achieved in the fluoxetine+Mg group, and the lowest was achieved in the TCA group. A comparison of these two groups showed a significant association between treatment type and treatment response *(p* = 0.029, Phi = 0.30). A comparison of patients receiving fluoxetine augmented with Mg ions and those receiving fluoxetine alone showed no significant association between the treatment type and treatment response (*p* = 0.445, Phi = 0.17).

Remission, defined as a decrease in the HDRS score to 6 or less after 8 weeks of treatment, was achieved in 9 patients (25.0%) from the TCA group, in 6 patients (22.2%) from the fluoxetine group, and in 6 patients (35.3%) from the fluoxetine + Mg group.

The groups did not differ significantly in terms of reaching a remission after 8 weeks of treatment (*p* = 0.615). A post hoc analysis revealed the highest remission rate achieved in the fluoxetine+Mg group. A pair comparison of this group with the TCA group and the fluoxetine group showed no significant associations (*p* = 0.519, Phi = 0.11 and *p* = 0.489, Phi = 0.14, respectively) between treatment type and remission after 8 weeks of treatment.

Antidepressant blood levels were assessed at all time points, with the purpose of maintaining drug levels within the therapeutic range [15,24,27], which was reached on day 14–21 of treatment.

The EEG patterns considered to constitute a positive pharmaco-EEG profile of antidepressants exhibited the following characteristics: increased power of beta, delta, and theta oscillations; decreased power of alpha oscillations; and slower alpha rhythms.

**Figure 1:**
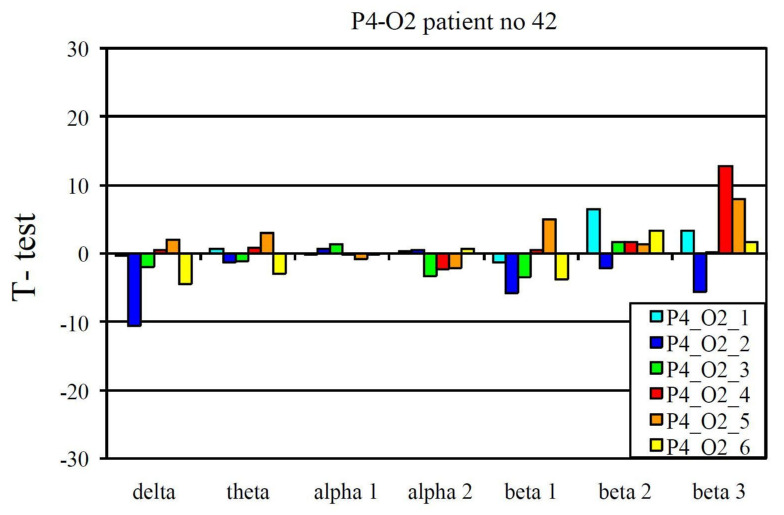
A positive pharmaco-EEG profile observed during tricyclic antidepressant treatment.

**Figure 2:**
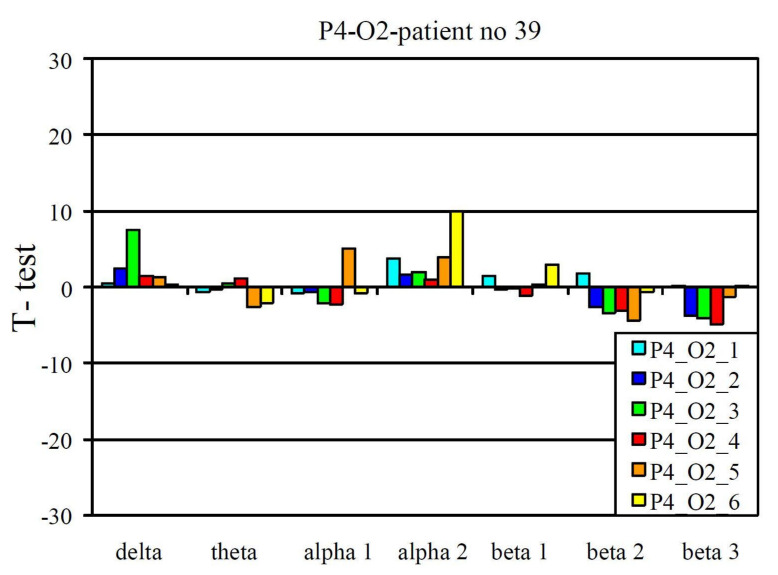
Example of a negative pharmaco-EEG profile during tricyclic antidepressant treatment.

The EEG profile presented in Figure 1 is an example of a positive pharmaco-EEG profile of antidepressants. The EEG profile presented in Figure 2 is an example of a negative pharmaco-EEG profile of antidepressants.

A pharmaco-EEG profile considered to reflect positive evidence of antidepressant effects in patients treated with fluoxetine alone and fluoxetine+Mg showed changes that were also characterized by increased power of beta, delta, and theta oscillations; decreased power of alpha activity; and slowed alpha rhythms.

**Figure 3:**
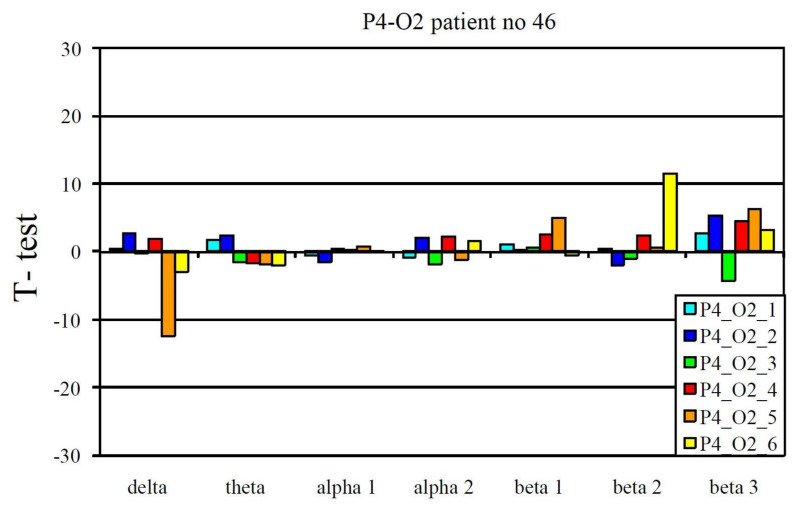
A positive pharmaco-EEG profile during selective serotonin reuptake inhibitor treatment.

**Figure 4:**
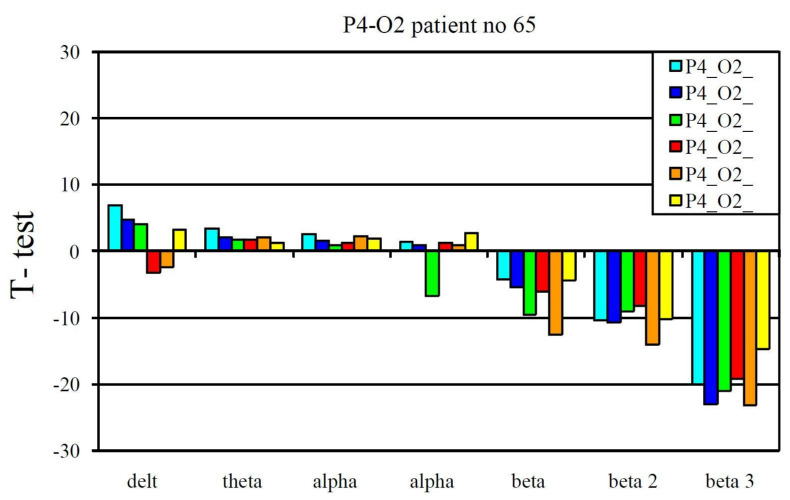
Example of a negative pharmaco-EEG profile during selective serotonin reuptake inhibitor treatment.

The EEG profile presented in Figure 3 is an example of a positive pharmaco-EEG profile of SSRIs. The EEG profile presented in Figure 4 is an example of a negative pharmaco-EEG profile of SSRIs.

Patient No. 65, who received fluoxetine, is an example of a patient with a pharmaco-EEG profile that showed no characteristics typical of antidepressant treatment.

The results observed in the TCA group were as follows:

– 6 h following antidepressant treatment initiation, 35.3% of patients achieved a positive pharmaco-EEG profile;
– 24–48 h after antidepressant treatment initiation, 30.3% of patients achieved a positive pharmaco-EEG profile;
– 14 days after treatment initiation, 55.9% of patients achieved a positive pharmaco-EEG profile;
– 4 weeks after treatment initiation, 63.6% of patients achieved a positive pharmaco-EEG profile;
– At week 6, 56.7% of patients achieved a positive pharmaco-EEG profile; and
– At week 8, 71.4% of patients achieved a positive pharmaco-EEG profile.

The fluoxetine group results were as follows:

– 6 h after antidepressant treatment initiation, 51.4% of patients achieved a positive pharmaco-EEG profile;
– 24–48 h after treatment initiation, 54.3% of patients achieved a positive pharmaco-EEG profile;
– 14 days after treatment initiation, 57.6% of patients achieved a positive pharmaco-EEG profile;
– At week 4, 46.7% of patients achieved a positive pharmaco-EEG profile;
– At week 6, 58.6% of patients achieved a positive pharmaco-EEG profile; and
– At week 8, 59.3% of patients achieved a positive pharmaco-EEG profile.

The results observed in the fluoxetine + Mg group were as follows:

– 6 h after antidepressant treatment initiation, 41.2% of patients achieved a positive pharmaco-EEG profile;
– 24–48 h after treatment initiation, 58.8% of patients achieved a positive pharmaco-EEG profile;
– 14 days after treatment initiation, 58.8% of patients achieved a positive pharmaco-EEG profile;
– At week 4 of treatment, 62.5% of patients achieved a positive pharmaco-EEG profile;
– At week 6, 70.6% of patients achieved a positive pharmaco-EEG profile; and
– At week 8, 62.5% of patients achieved a positive pharmaco-EEG profile.

**Figure 5:**
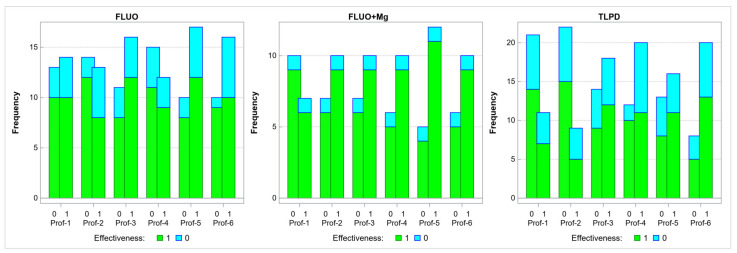
The number of patients who achieved (1) and failed to achieve (0) a positive pharmaco-EEG profile at the individual time points in three treatment groups, with green areas indicating a 50% improvement in the HDRS scores at week 8. FLUO—fluoxetine; Mg—magnesium; TLPD—tricyclic antidepressant; Prof—pharmaco-EEG profile; and Effectiveness—adequate treatment response.

Each panel of Figure 5 represents one of the evaluated treatments and shows pairs of vertical bars representing the number of patients who achieved (1) and failed to achieve (0) a positive pharmaco-EEG profile at the consecutive time points. The green area in each bar represents the number of patients who achieved a 50% improvement in their HDRS scores at week 8 of treatment, and the blue area represents the number of patients who failed to achieve such improvement.

An analysis of the fluoxetine group results showed no significant association between the presence of positive pharmaco-EEG profiles and achieving a 50% improvement in the HDRS score at any study time point.

An analysis of the fluoxetine+Mg group results showed no significant association between positive pharmaco-EEG profiles and achieving a 50% improvement in the HDRS score at any study time point.

An analysis of the TCA group results showed no significant association between positive pharmaco-EEG profiles and achieving a 50% improvement in the HDRS score at any study time point.

**Figure 6:**
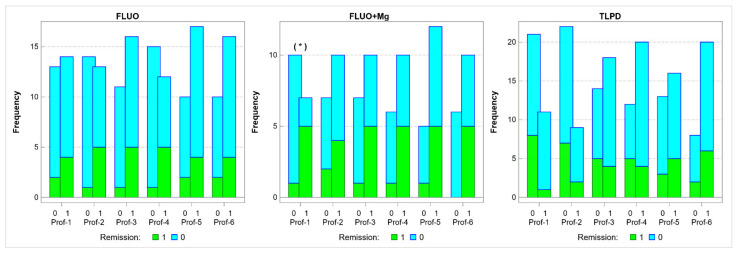
The number of patients who achieved (1) and failed to achieve (0) a positive pharmaco-EEG profile at the individual time points in three treatment groups, with green areas indicating HDRS remission at week 8. FLUO—fluoxetine; Mg—magnesium; TLPD—tricyclic antidepressant; Prof—pharmaco-EEG profile; and * *p* < 0.05.

Figure 6 presents the data from the three treatment groups at all time points. The pairs of vertical bars represent the numbers of patients who achieved (1) and failed to achieve (0) a positive pharmaco-EEG profile at each time point. The green area in each bar represents the number of patients who achieved remission based on their HDRS scores at week 8, and the blue area represents the number of patients who failed to achieve remission.

An analysis of the fluoxetine + Mg group results showed a significant association between a positive pharmaco-EEG profile at the 6-h time point and achieving HDRS remission at week 8 (*p* = 0.035, Phi = 0.63).

No significant association between a positive pharmaco-EEG profile and HDRS remission was observed at any other time points.

An analysis of the fluoxetine group results showed no significant association between positive pharmaco-EEG profiles and achieving HDRS remission at any time point.

An analysis of the TCA group results showed no significant association between positive pharmaco-EEG profiles and achieving HDRS remission at any time point.

## 4. Discussion

Since the year 1992, the WUM Department of Psychiatry has been evaluating the use of therapeutic drug monitoring (TDM) in clinical practice [15,27]. Our TDM studies involve thorough assessments of the patients’ condition with the use of widely available psychometric scales, antidepressant drug serum level monitoring, and evaluation of any treatment-related changes in EEG patterns. Most of the available reports on the use of TDM in clinical practice focused on a more limited range of monitored parameters (with typically only two parameters being assessed).

The duration of clinical studies involving antidepressant drugs is usually too short (6 weeks in total), most likely on economic grounds.

An adequate treatment response has been defined as a reduction in the baseline HDRS score by at least 50%, which is interpreted as achieving a therapeutic effect.

In recent years, the term “adequate response to treatment” has been often replaced by the term remission, i.e., a symptom-free period. This term is meant to describe effective antidepressant treatment [30].

In our study, the highest response rates were observed in the group on the fluoxetine+Mg regimen whereas the lowest response rates were observed in the TCA group. This difference was statistically significant.

Zinc and magnesium are potent inhibitors of the NMDA receptor complex. Studies involving a model of depression in rodents showed that zinc and magnesium have antidepressant effects similar to those of receptor NMDA antagonists.

Experimentally induced hypomagnesemia leads to anxiety- and depression-like behaviors in laboratory animals. There is evidence of magnesium ions playing a role in the treatment of affective disorders. The effect of magnesium on the hypothalamic-pituitary-adrenal (HPA) axis, the suppression of hippocampal kindling, the modulation of adrenal cortical sensitivity, and a direct effect on the function of the P-glycoprotein transporter in the blood–brain barrier may explain the pathogenesis of depressive disorders [31].

The antidepressant and anxiolytic effects of magnesium have been observed after both short-term and long-term magnesium administration. These effects depend on magnesium levels in blood. No development of tolerance was observed [32].

The use of a magnesium-poor diet (10% of the daily recommended dose) in mice over several weeks yielded increased depressive behaviors. These behaviors were successfully reversed with antidepressant and anxiolytic drugs [33].

Mice receiving magnesium-poor diet (10% of the daily recommended dose) were shown to be more predisposed to exhibit depressive behaviors responding to antidepressant treatment [34].

Magnesium deficiency in mice may have an adverse impact on HPA axis activity. Long-term desipramine treatment reverses the observed HPA axis dysfunction. Magnesium deficiency in mice results in an anxiety responsive to antidepressant and anxiolytic drugs. Some authors suspect that HPA axis dysfunction may contribute to emotional hyper-reactivity in response to a magnesium-poor diet [35].

Poleszak et al. evaluated the impact of magnesium on the effects of antidepressant drugs with various mechanisms of action (reboxetine and tianeptine) in the mouse forced swim test (FST) [32,36]. Experimental and epidemiological studies suggest that disturbances in magnesium metabolism are present in affective disorders [31,37]. In animals, magnesium deficiency leads to a reduction in offensive behavior and to an increase in defensive behavior [38]. Moreover, magnesium administration reduces immobility time in the FST in mice [39].

Botturi [40] reported the efficacy of magnesium supplementation in the treatment of depression.

We do not have any direct data indicating that a combined magnesium and fluoxetine treatment might have a trophic effect.

The glutamatergic theory of depression is also associated with the participation of the BDNF. Current data reveal that blood BDNF levels increase during treatment with antidepressants. It has been proven that BDNF results in a decrease in the expression of NMDA receptor subunit mRNA [41,42].

Based on the evidence that a combined imipramine and zinc treatment enhances brain BDNF levels [43], we can assume a similar effect is achieved with a combination of magnesium and fluoxetine, which of course should be investigated experimentally as part of a prospective, randomized, placebo-controlled study.

Fluoxetine may also influence magnesium metabolism/distribution. The enhanced effects of fluoxetine administered in combination with oral Mg suggest common mechanisms of action.

Fluoxetine increases the survival of hippocampal neurons by improving axonal transport in a stress-induced model of depression in male rats [41]. The antidepressant-like effect of magnesium in the olfactory bulbectomy model is associated with the AMPA/BDNF pathway [42]. A review of the literature yielded no reports on antidepressant treatment potentiation with magnesium ions or its effects on the pharmaco-EEG profile.

Pharmaco-EEG is a noninvasive method used to evaluate the effect of pharmaceutical compounds on the central nervous system by analyzing EEG signals, which, directly and in high resolution, represent spontaneously synchronized postsynaptic potentials in the cerebral cortex. The recently published International Pharmaco-Encephalography Society (IPEG) guidelines, established by a panel of EEG experts, aim to help standardize pharmaco-EEG in humans and to facilitate comparability of data across laboratories, thus making it possible to pool data and to conduct meta-analyses [44]. The recommended standard experimental EEG protocol involves measuring wake and sleep EEG activity. The introduction of fluvoxamine and fluoxetine in the 1980s, and the subsequent introduction of other SSRIs caused a growing interest in their effect on EEG patterns.

The basic assumptions behind pharmaco-EEG are that all behavior-modifying chemical compounds also affect EEG patterns [11,12,13,14], that the directionality of EEG changes depends on the clinical effects of the drug, that drug-induced changes in EEG are the same in healthy people and in patients, and that short-term changes in EEG are predictive of the changes occurring over long-term treatment [11,15,21,22].

SSRI studies conducted by Saletu showed that a single fluoxetine dose of up to 60 mg produces a weak desipramine-like effect whereas a 75 mg dose produces an imipramine-like effect [20].

EEG changes produced by sertraline at 100 mg resembled those observed with desipramine, whereas at doses of 200 mg and 400 mg, the changes resembled those observed with imipramine [19]. Knott demonstrated that a single dose of paroxetine produced no changes in EEG. However, after 6 weeks of treatment, they observed a decreased power of alpha oscillations and an increased power of delta, theta, and beta oscillations (imipramine-like pattern) [45].

Saletu’s studies from the 1980s and 1990s attempted to classify SSRI (in a way similar to the classification of TCAs) into thymoleptic agents (imipramine- and amitriptyline-type drugs)—this group includes fluvoxamine [16,17], venlafaxine [18], and sertraline at doses above 100 mg [19]—and tachythymoleptic agents (desipramine-type drugs)—such as fluoxetine [16,20] and sertraline at doses under 100 mg [19].

Tarn and Kwon observed no EEG changes after 4 and 6 weeks of fluoxetine treatment [46,47].

Earlier studies conducted at the WUM Department of Psychiatry showed that achieving a pharmaco-EEG profile of antidepressants at an initial stage of treatment (6 h after administration of the first dose) had a significant predictive value in estimating the response to treatment achieved 8 weeks later [15].

The fluoxetine+Mg group in our present study showed a significant association between a positive pharmaco-EEG profile 6 h after administration of the first dose and achieving remission after 8 weeks of treatment.

The studies that were conducted at the WUM Department of Psychiatry in Warsaw over the period of many years [11,28] show promise in terms of the usefulness of pharmaco-EEG as a tool for early assessment of the effect of treatment and adjusting the dose of a particular drug to accommodate the needs of the given patient via TDM.

A systematic meta-analysis of 201 articles assessing the effect of psychotropic drugs on EEG patterns indicated that, despite the growing body of knowledge on this topic, further studies in larger groups of patients are needed to confirm previous observations in order to allow a clinical correlation of the patient’s response to a given drug to be made with that of a drug’s effects on EEG patterns [48].

Due to the relatively low cost of treating depression with antidepressants augmented with magnesium and the low cost of pharmaco-EEG, these studies should be continued. The observations from the present study require confirmation in a larger group of patients.

## 5. Conclusions

The highest rate of adequate treatment response (88.2%) was observed in the fluoxetine+Mg group, whereas the lowest rate (58.3%) was observed in the TCA group. A comparison of these two groups revealed a significant association between the type of treatment and treatment response (*p* = 0.029, Phi = 0.30).

The fluoxetine+Mg regimen data showed a significant association between the presence of a positive pharmaco-EEG profile 6 h after treatment initiation and achieving remission (based on the HDRS score) at week 8 (*p* = 0.035, Phi = 0.63).

No significant association was observed between the character of pharmaco-EEG profile and the response to treatment.

The potentiating effects of magnesium ions may offer an alternative to the standard treatment for depression.

Our observations need to be confirmed in a prospective, randomized, placebo-controlled study involving a larger group of patients.

## Data Availability

The data are available from the corresponding author upon request.
